# Dissecting the roles of the Tuberin protein in the subcellular localization of the G2/M Cyclin, Cyclin B1

**DOI:** 10.1371/journal.pone.0272741

**Published:** 2022-08-10

**Authors:** Adam Pillon, Jessica Dare-Shih, Jackie Fong, Elizabeth Fidalgo da Silva, Lisa A. Porter

**Affiliations:** Department of Biomedical Sciences, University of Windsor, Windsor, Ontario, Canada; University of Rome, ITALY

## Abstract

Tuberin is a major component of the protein regulatory complex known as the Tuberous Sclerosis Complex and plays a crucial role in cell cycle progression and protein synthesis. Mutations in the Tuberin gene, *TSC2*, lead to the formation of benign tumors in many organ systems and causes the Tuberous Sclerosis Complex disorder. Genotypes ranging from point mutations to large deletions in the *TSC2* gene have been clinically characterized with a wide range of phenotypes from skin tumors to large brain tumors. Our lab has previously demonstrated that Tuberin can directly bind and regulate the timing of nuclear transport of the G2/M cyclin, Cyclin B1. Herein we study the consequence of one clinically relevant truncation in the Tuberin protein on cell cycle function. We demonstrate that exogenous expression of a fragment of the N-term region of Tuberin alters the subcellular localization of Cyclin B1 and increases cell proliferation. This adds to our body of information about the residues within Tuberin responsible for regulating the cytoplasmic retention of Cyclin B1 and supports the phenotypic data seen in the clinic with Tuberous Sclerosis Complex patients harbouring similar large deletions in Tuberin.

## Introduction

**Fig 1:**
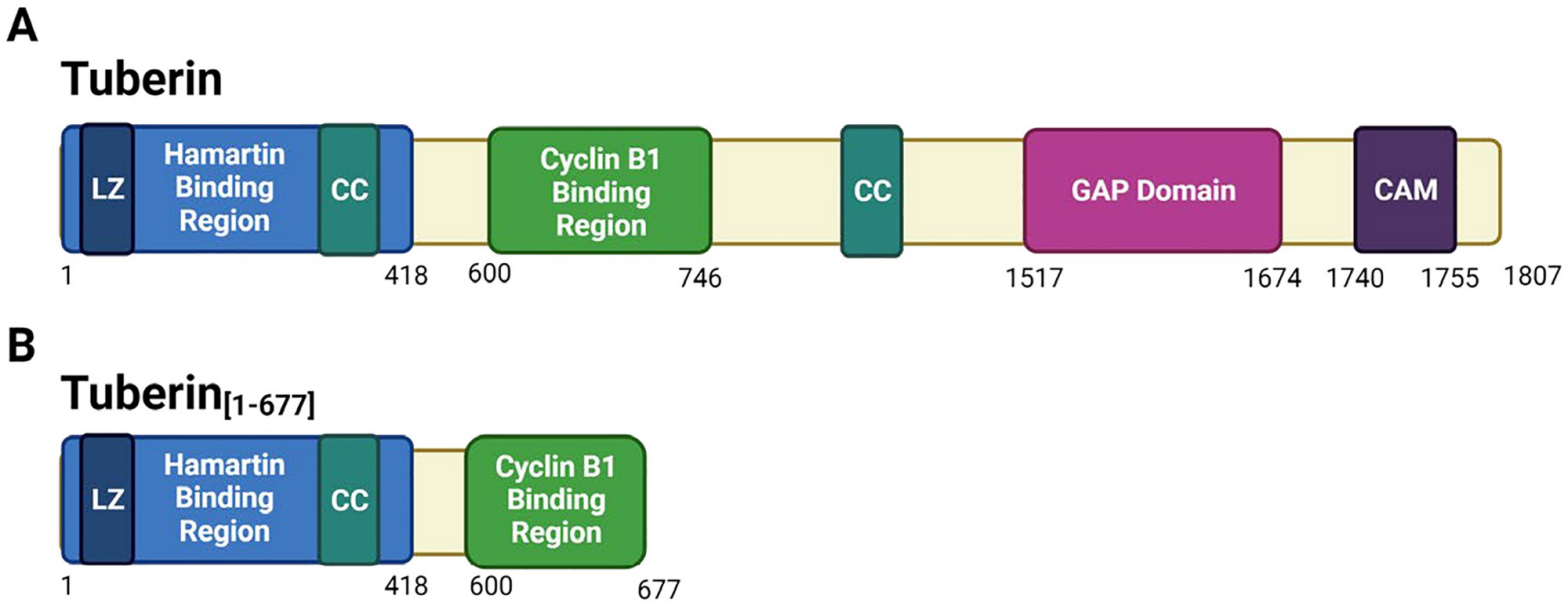
Simplified protein structure diagram of the Tuberin protein and Tuberin_[1–677]_. The primary structure of Tuberin (A: Top Panel) and Tuberin_[1–677]_ (B: Bottom Panel) showing domains and binding patterns. The numbers below the diagram are the amino acids where each domain region starts and ends. LZ (Leucine zipper), CC (Coiled-coiled domain) and CAM (Calmodulin binding domain) are depicted. Image created with BioRender.com.

The Tuberin protein is encoded by the *TSC2* gene located on chromosome 16p13.3 consisting of 41 exons. It is comprised of 1804 amino acids and results in a 200 kDa protein [1]. This large protein contains a variety of conserved domains responsible for its function: two coiled-coil domains (aa346-371 and 1008–1021), a leucine zipper (aa81-98), a GTPase activating protein (GAP) homology domain (aa1517-1674) and a calmodulin domain (aa1740-1758) [2] (Fig 1A). It is well-established that signals from the environment, including nutrient status, can alter the phosphorylation, protein-protein interactions and subcellular localization of Tuberin which are integrated to impact aspects of cell physiology [3–5]. The most well-known effector of Tuberin being the regulation of cell growth and protein synthesis through the downstream inhibition of the Mammalian Target of Rapamycin (mTOR) [5, 6]. The Tuberin GAP domain inactivates mTOR signalling by stimulating auto-hydrolysis of Rheb-GTP, converting it to Rheb-GDP [4].

In addition to these roles, Tuberin also directly regulates the cell cycle, inhibiting progression through G1/S and G2/M under select environmental conditions [7–9]. During G1 phase of the cell cycle Tuberin, independent of Hamartin, interacts with the Cyclin-Dependent Kinase (CDK) inhibitor, p27, protecting p27 from ubiquitin-mediated degradation and permitting the nuclear accumulation of p27 by interfering with the 14-3-3β dependent cytoplasmic retention of p27 [7]. Through this mechanism, Tuberin markedly inhibits the G1/S transition [10]. In addition to inhibitory roles at the G1/S transition, Tuberin forms a transient complex with Cyclin B1 (CycB1) at the G2/M transition, providing a novel mechanism which CycB1 localization and mitotic onset is regulated. Tuberin-CycB1 complex formation is in part regulated by the phosphorylation on five serine residues within the Cytoplasm Retention Signal (CRS) region of CycB1. Tuberin has lower affinity to CycB1 when these serine residues are mutated to a glutamic acid to mimic phosphorylation (CycB1-5xE), and a higher affinity for binding CycB1 where these sites are altered to non-phosphorylatable alanine residues (CycB1-5xA) [8]. The formation of the Tuberin-CycB1 complex is dependent on serum levels, whereby ample nutrients permits complex formation that subsequently supports a delay in mitotic progression and an increase in cell size [9]. The Tuberin-CycB1 complex also associates with both Hamartin and the G2 CDK, CDK1. Interestingly, CDK1 has been shown to phosphorylate Hamartin in the Tuberin binding domain to aid in the inhibition of pS6Kinase during the cell cycle [11]. Collectively, these data present the possibility that feedback from the Tuberin-CycB1 complex can be provided to regulate G1/S. Hence, Tuberin communicates information about the levels of nutrients, growth factors, and energy stores to the cell cycle machinery to regulate cell division [12–14].

The syndrome Tuberous Sclerosis Complex (TSC) is characterized by the formation of benign tumors, referred to as hamartomas, that occur in a variety of organ types such as the brain, heart, skin, kidney, and lungs [2, 13, 15]. TSC mutations most frequently occur during development and the syndrome presents itself in early childhood [16]. Traditionally, TSC is considered an autosomal-dominate disorder acquired through inheritance; however, approximately two thirds of TSC cases arise from sporadic germ-line mutations. Disease prevalence affects approximately 1 in 6000 live births annually and is estimated to affect roughly 1.5 million individuals worldwide [15, 17, 18]. TSC can arise from mutations in both the *TSC1* and *TSC2* genes; however, mutations in *TSC2* result in more severe disease related phenotypes [18–20]. Deletion, truncation, or mutation in *TSC2* can result in severe cellular outcomes [21, 22]. The tremendous variability in phenotypes seen from alterations in TSC is accredited to the fact that mutations in *TSC2* span the entire length of the gene. Understanding the cellular consequences of different mutations/deletions is necessary to fully understand the overall phenotype and to identify treatment needs for individual patients.

Protein truncation test (PTT) detects changes in the DNA that can cause premature protein termination. This test has shown that truncation of the Tuberin protein is a common event in TSC patients [18]. From 18 patients analyzed by PTT, six showed altered size of the Tuberin protein [23]. Single-strand conformational polymorphism (SSCP) analysis [24] has showed three TSC patients from a total of 30 presented TSC2 sequence changes predicting a truncated protein [25]. Sequencing analysis revealed a novel Tuberin truncation mutant that arises from a deletion at exon 24 in the *TSC2* gene [26]. This mutation results in a 946 amino acid protein, which is expected to retain the ability to bind Hamartin. Another Tuberin truncation protein containing only the amino acids 1–607 is still able to bind Hamartin [27]. These truncation proteins lack the C-terminal GAP domain, transcriptional activation domains, and many critical phosphorylation sites. Naturally, it can be expected that loss of these critical domains in the endogenous protein would prevent the tumor suppressor function of Tuberin and lead to uncontrolled growth resulting in tumor initiation.

Herein we analyze the effects of a truncation mutant of *TSC2* gene, Tuberin _[1–677],_ containing the Hamartin binding region and a portion of the CycB1 binding region, similar to the previously identified clinical mutations mentioned above. Tuberin_[1–677]_ lacks the GAP domain and many of the phosphorylation sites responsible for nutrient/growth factor regulation. Our data shows that the truncated protein has a decreased ability to retain CycB1 in the cytoplasm and despite the presence of endogenous Tuberin protein increases cell proliferation and mitotic onset.

## Material and methods

### Plasmids

pCMV-Tag2-Mock and full-length human pCMV-Tag2-*TSC2* mammalian expression vectors (Flag-tagged) were supplied by J. DeClue. Human full-length CycB1-WT in a pCMX backbone (GFP tagged) were generous gifts from J. Pines and have been previously described [28]. Tuberin_[1–677]_ was constructed using site-directed mutagenesis by the insertion of a stop codon at the position 2050 of the *TSC2* cDNA inserted into the pCMV-Tag2-*TSC2* vector.

### Cell culture

HEK-293 cells (ATCC) were maintained in Dulbecco’s Modified Eagle Medium (DMEM) supplemented with 10% or 0.5% fetal bovine serum (FBS; Gibco) and 1% Penicillin- Streptomycin (Thermo-Fisher). Cells were incubated at 37°C in 5% CO2.

### Antibodies

The following antibodies were utilized during Western Blotting and indirect immunofluorescent analysis: Mouse α-CycB1 (monoclonal; Santa Cruz; Cat# H1809), mouse α-FLAG (Sigma; Cat# F1804), rabbit β3-Tubulin (monoclonal; NEB; Cat#5568), goat α- mouse IgG (Sigma; Cat# A4416), goat α-rabbit IgG (Sigma; Cat#A6154), Alexa 488- conjugated α- rabbit IgG (Invitrogen; Cat# A-11008), Alexa 568- conjugated α- mouse IgG (Invitrogen; Cat# A-11004), rabbit α-phospho-H3(S-10) (ABcam); Cat# ab32107), Alexa Fluor 488 Phalloidin (Thermo Fisher; Cat# A12379).

### Transfection and immunoblotting

Transfection and immunoblotting have been previously described [9]. Briefly sub-confluent HEK-293 cells were transfected with branched PEI (Polysciences, Inc.) 18 to 24 hrs after transfection the cells were lysed or washed with 1X PBS and cultured in normal serum medium (10% FBS) or low serum medium (0.5% FBS) for 24 hrs previous to the lysis. The lysed samples were resolved by 10% SDS-PAGE. The membrane was blotted with the indicated antibody (1:1000) followed by enhanced chemiluminescence (ECL) (Amersham). Chemiluminescence was quantified on a ChemiDoc MP V3 (BioRad) and densitometry performed using AlphaView SA software. Densitometry is performed by obtaining the total densitometry units of each band. Bands looking at total protein levels (lysate blots) are normalized to the β-tubulin loading control band in the corresponding lane.

### Immunofluorescent microscopy

Cells were seeded onto glass coverslips and transfected as described above. 18 hrs following transfection, cells were fixed with 4% formaldehyde in phosphate-buffered saline for 20 minutes and permeabilized with 0.02% Triton X-100 for 5 min. Primary antibodies were used at 1:500, secondary antibodies at 1:1300, and Phalloidin stain at 1:20. Hoechst stain (Sigma) was added to the permeabilizing solution to a final concentration of 0.5μg/ml. Slides were imaged by fluorescence microscopy using Zeiss Blue Confocal microscopy with 20x lens and analysed by the accompanying software.

### Cycloheximide chase assay

HEK-293 cells were seeded onto 6 cm plates and transfected as described above. 18 hours following transfection the media was aspirated, the cells were washed with 1mL of 1xPBS and 3mL of media containing 100μM of cycloheximide diluted from 1mg/mL stock (Sigma, Cat# C4859) was added to the plate. The cells were incubated for up to 6 hours in the cycloheximide media. Every two hours, the cells were collected and subjected to lysis buffer as described above.

### Proliferation assay

HEK-293 cells were seeded onto 6 cm plates and transfected as described above. 16 hours following transfection, the media was removed, and the cells were washed in 1mL of 1xPBS. Fresh media was added to each plate and the cells were incubated for 24, 48 or 72 hours. At each time point the cells were collected and counted using Trypan Blue to count alive cells. The remaining cells were subjected to cell lysis as described above to prepare for Western blotting.

## Results and discussion

To analyze the effects of large deletions in Tuberin on cell proliferation and localization of Cyclin B1, a truncated form of Tuberin was created by inserting a stop codon at the position 2050 into *TSC2* cDNA in the pCMV-tag2-*TSC2* vector. The Tuberin_[1–677]_ protein contains amino acids 1–677 which comprise the Hamartin binding region and a fragment of the CycB1 binding domain (Fig 1B).

**Fig 2:**
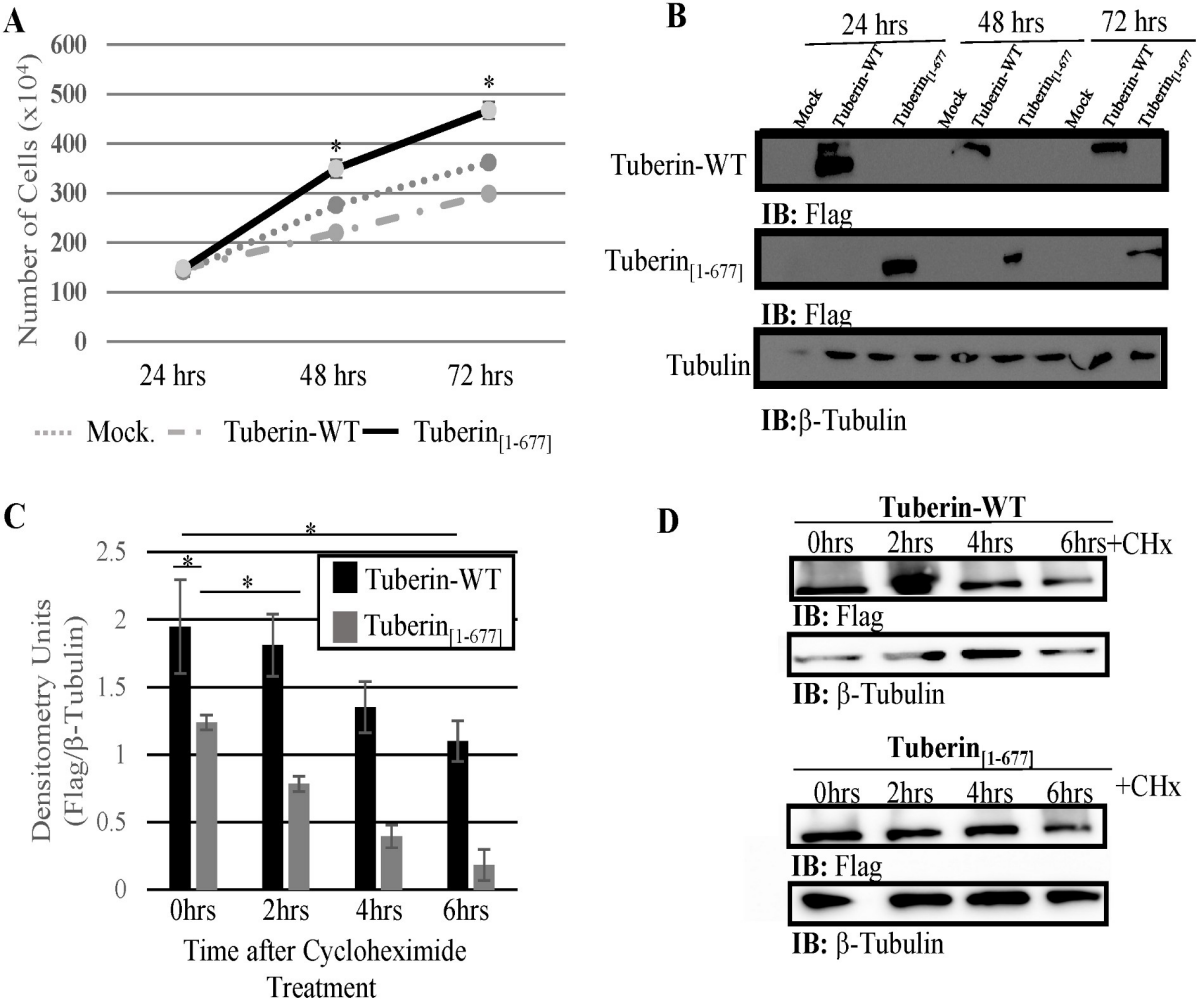
Tuberin_[1–677]_ overexpression significantly increased cell proliferation when compared to Tuberin-WT and control transfected cells. HEK-293 cells were seeded in equal numbers and transiently transfected for 24 hours with mock, Tuberin-WT or Tuberin_[1–677]_ expression vectors. **(A)** Cell numbers counted via trypan blue exclusion assay at 24, 48 and 72 hrs after transfection. Total number of alive cells are depicted. **(B)** Tuberin-WT and Tuberin_[1–677]_ expression was confirmed by Western blot using Flag antibody to detect the presence of the transfected proteins and β-Tubulin was used as a loading control. **(C)** Protein levels following cycloheximide treatment. *p<0.05. Statistical significance was assessed using students’ unpaired t-test. **(D)** Tuberin-WT and Tuberin_[1–677]_ expression was confirmed by Western blot using Flag antibody to detect the presence of the transfected proteins and β-Tubulin was used as a loading control. Blots are representative of three experiments.

Equal numbers of sub-confluent HEK-293 cells were transfected with mock, Tuberin-WT or Tuberin_[1–677]_ expression vectors. A time course proliferation assay was performed where equal numbers of transfected HEK-293 cells were collected at 24, 48, and 72 hours pos-transfection. Trypan blue exclusion was used to determine the number of live cells at each time point. Tuberin_[1–677]_ showed significantly higher numbers of cells over the time course when compared with mock or Tuberin-WT (Fig 2A). The proliferation assay demonstrates that despite the presence of endogenous Tuberin, Tuberin_[1–677]_ overexpression dramatically increases the number of alive cells as compared to Tuberin-WT or mock control. The overexpression of Tuberin-WT and Tuberin_[1–677]_ was confirmed through Western blot (Fig 2B). A cycloheximide assay was done to check the stability of Tuberin_[1–677]_ and it shows a significant decrease in the stability of the truncated protein when compared with Tuberin-WT. While Tuberin-WT level decreased to 44% the truncated protein presents a decrease of 86% in the protein level (Fig 2C and 2D). The level of expression of Tuberin_[1–677]_ is significantly less than Tuberin-WT without cycloheximide treatment (Fig 2B and 2C—0 hours) despite being driven from the same CMV promoter. Tuberin stability is in part controlled by AKT phosphorylation and methylation in the C-term domain of this protein [29, 30]. The lack of these regulatory domains in the truncated protein can be responsible for the different level of stability between Tuberin-WT and the truncated protein.

**Fig 3:**
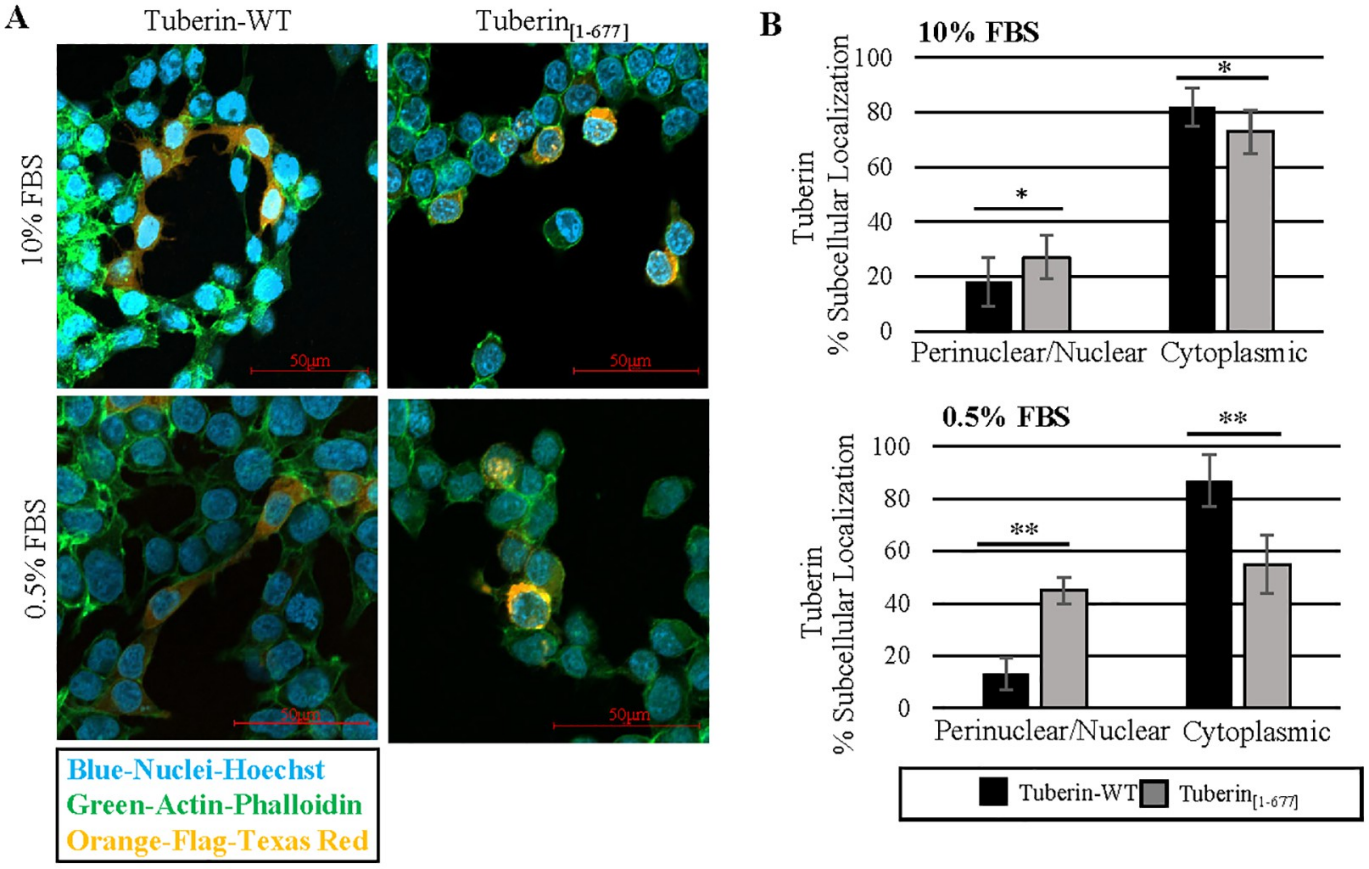
Tuberin_[1–677]_ exhibits perinuclear/nuclear localization significantly higher than Tuberin-WT. **(A)** HEK-293 cells were transiently transfected with Tuberin-WT or Tuberin_[1–677]_ expression vectors. After 18–20 hrs the cells were subjected to either 10% FBS (top panel) or low nutrient conditions, 0.5% FBS (bottom panel). For full panel see S1 and S2 Figs for grayscale images. **(B)** Cell counting for Tuberin-WT and Tuberin_[1–677]_ protein localization in 10% FSB (top panel) or 0.5% FBS (bottom panel). *p<0.05, **p<0.01. Statistical significance was assessed using a student’s unpaired t-test. Images are representative of three independent experiments.

The subcellular localization of Tuberin is regulated, at least in part, by the Calmodulin binding-domain (CaM) which overlaps with a NLS (nuclear localization sequence) and the AKT/p90 ribosomal S6 kinase-1 and AKT phosphorylation sites located in the C-terminal of Tuberin which have been linked to nuclear localization [31, 32]. To determine the subcellular localization of Tuberin_[1–677]_, the truncated protein and Tuberin-WT were overexpressed in HEK-293 cells and the cellular localization of these proteins in 10% or 0.5% serum (FBS) were analyzed via immunofluorescence microscopy (Fig 3; S1 and S2 Figs). Tuberin-WT was localized diffusely throughout the cytoplasm as we and others have previously demonstrated [8]. The truncated protein however was localized to the nucleus or as a circular staining pattern surrounding the nucleus as compared to the Phalloidin staining cytoplasmic F-Actin, often referred to as perinuclear (Fig 3B). This effect was enhanced in reduced serum conditions (Fig 3B: Lower panel).

**Fig 4:**
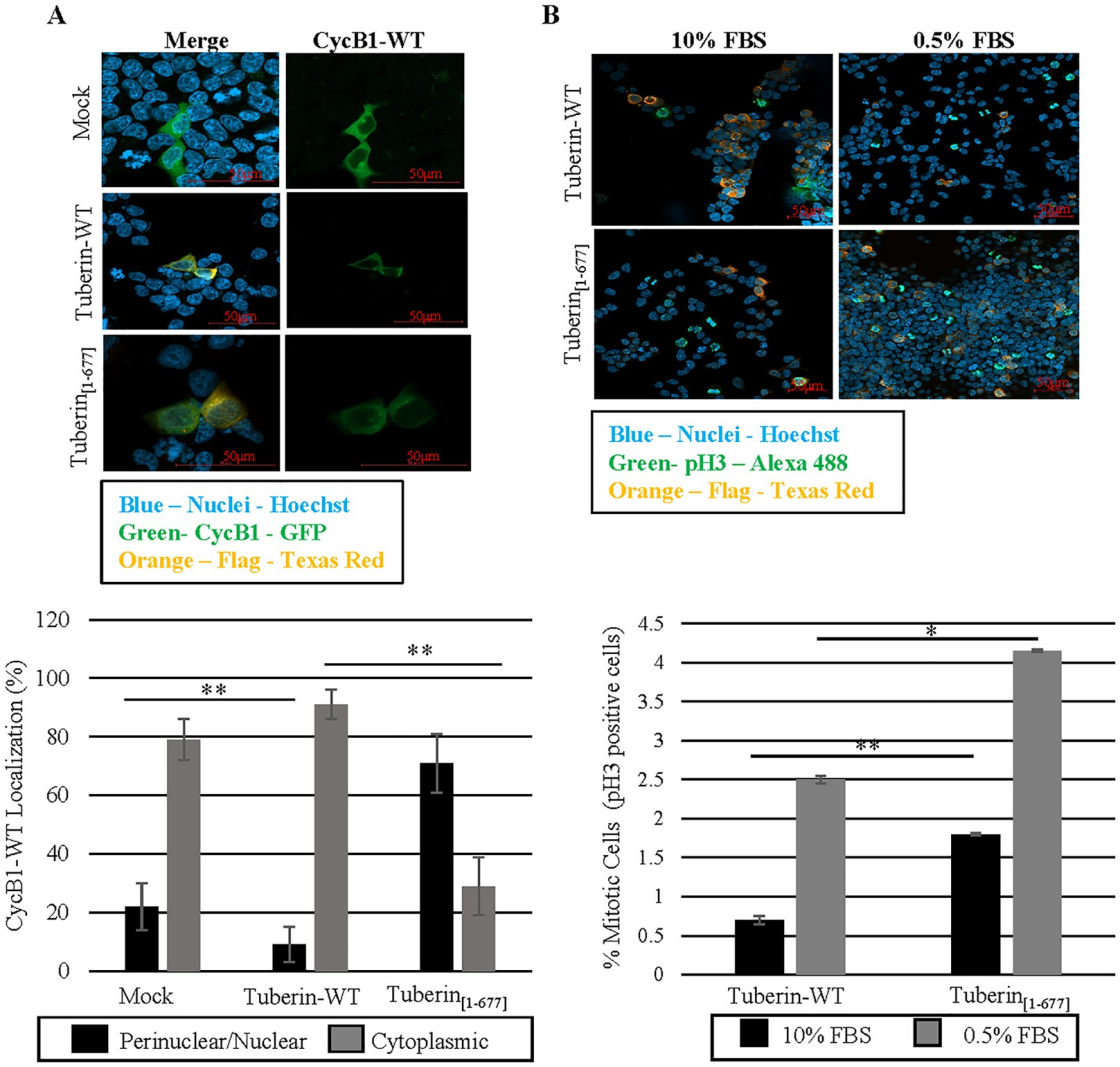
Sub-cellular localization of CycB1 and the mitotic onset are regulated differently by Tuberin-WT and Tuberin_[1–677]._ **(A)** HEK-293 cells were transiently transfected with control (mock), Tuberin-WT or Tuberin_[1–677]_ and CycB1-WT-GFP expression vectors and immunofluorescence was conducted 18–20 hrs pos-transfection. Immunofluorescence images (top panel). For full panel and blot confirming protein expression see S3 Fig. Cells were counted for their respective CycB1-WT-GFP localization and compared to total number of transfected cells (bottom panel). **(B)** pH3 was used as a marker for mitotic index in cells transfected with Tuberin-WT or Tuberin_[1–677]_ under normal (10%) or low (0.5%) serum conditions (top panel). Cells were counted for pH3 staining in cells expressing Tuberin-WT or Flag-Tuberin_[1–677]_ and compared to total number of transfected cells (bottom panel). For full panel and blot confirming protein expression see S4 Fig. *p<0.03 **p<0.01. Statistical significance was assessed using student’s unpaired t-test. Images are representative of three experiments.

It has been demonstrated AKT phosphorylation of Tuberin at S939 increases Tuberin binding to the G2/M Cyclin, CycB1, prior to phosphorylation of CycB1 in the CRS region [9]. Reduced, but not abrogated binding between CycB1 and Tuberin occurs with CycB1 phosphorylation in the CRS region and the onset of mitosis when Tuberin/CycB1 complex localizes in the nucleus [8]. To determine the effects of Tuberin_[1–677]_ on CycB1 localization immunofluorescence microscopy was done in cells co-overexpressing Tuberin_[1–677]_ or Tuberin-WT (Fig 4A; S3 Fig). Consistent with previous literature [8], nearly 90% of CycB1 was found to be retained in the cytoplasm when co-expressed with Tuberin-WT. Interestingly, when CycB1 is overexpressed with Tuberin_[1–677]_, the subcellular localization is altered and is significantly more perinuclear/nuclear CycB1 than Tuberin-WT or cells overexpressing only CycB1-WT (mock) (Fig 4). Hence, Tuberin_[1–677]_ lacks the regulatory domains/phosphorylation sites responsible for retaining CycB1 in the cytoplasm and is able to actively traffic CycB1 to the perinuclear/nuclear space.

Tuberin-WT delays mitotic onset by retaining CycB1 in the cytoplasm, a molecular function which depends on serum levels [8, 9]. The mitotic index was assessed using pH3 staining as a mitotic marker to verify if Tuberin_[1–677]_ can control the mitotic onset as Tuberin-WT (Fig 4B; S4 Fig). Overexpression of Tuberin-WT indeed decreases the mitotic index when the cells are cultured in 10% FBS and no significative effect is observed at low serum condition (0.5% FBS) when comparing with the control as previously described [8, 9]. Interestingly, there is a significant increase in the mitotic index when the cells are expressing Tuberin_[1–677]_ as compared to Tuberin-WT, this is seen in both 10% and 0.5% FBS. These results confirm the high proliferation rate observed when cells are expressing Tuberin_[1–677]_ (Fig 2A).

Tuberin mutations are a large contributing factor to misregulated cell growth pathways, supporting abnormal cell proliferation and hamartoma formation [33–37]. The results presented here indicate that a clinically relevant truncation of the Tuberin_[1–677]_ protein has lost the ability to act as a tumor suppressor controlling the G2/M cell cycle transition and actively causes an increase in cell proliferation. Collectively, this work adds to our understanding of the function of Tuberin in regulating the sub-cellular localization of CycB1 and regulating the onset of mitosis, while providing an observational analysis of a possible functional problems of TSC truncation mutations.

These data may be relevant and important with respect to disease pathogenesis and is the first of its kind to assess a Tuberin truncation mutant *in vitro*. Most of the treatments available to TSC patients focus on inhibition of the mTOR protein synthesis pathways [38]. This work adds to the growing body of data supporting that it is important to understand the full scope of the biology for both Tuberin-WT regulation and that of clinical mutations and that this may result in novel treatment approaches. Additionally, this work demonstrates that imbalances in the levels of Tuberin mutants, including amplifications, can cause adverse consequences for cell biology.

## Conclusion

This is the first study to assess how this specific truncation of the Tuberin protein may contribute to disease manifestation. We show that elevated levels of the truncation mutant Tuberin_[1–677]_ lacking the inhibitory GAP domain retains the ability to regulate the localization of the G2 cyclin, CycB1. We further show that this supports increased levels of mitosis and an increase in overall cell proliferation. This work stresses that understanding the biology of different mutations/deletions in this protein is important for understanding normal and abnormal physiology.

## Supporting information

S1 Fig Subcellular localization of Tuberin and Tuberin_[1–677]_ under normal and low nutrient conditions. HEK-293 cells were transiently transfected with Tuberin WT or Tuberin_[1–677]_ expression vectors on plates containing coverslips. After 18–20 hrs the cells were subjected to either 10% FBS (normal nutrient–panel A) or 0.5% FBS (low nutrient–panel B) conditions for 24 hrs. Cells were collected for lysate and coverslips were subjected to immunofluorescence protocol describe in Material and Methods. FLAG-Texas Red (red) labelling cells expressing Tuberin or Tuberin_[1–677]_, Hoechst (blue) as a nuclei marker, and FITC-Phalloidin (green) to stain F-Actin.
(TIF)

S2 Fig Subcellular localization of Tuberin and Tuberin_[1–677]_ under normal and low nutrient conditions. (A, B) in grayscale. Images from S1 Fig were transformed to grayscale in to provide a better visual contrast in the subcellular localization of the proteins in study.
(TIF)

S3 Fig Subcellular localization of Cyc B1 WT when co-transfected with Tuberin or Tuberin_[1–677]._ HEK-293 cells were transiently transfected with control (mock), Tuberin-WT or Tuberin_[1–677]_ and CycB1 WT-GFP expression vectors on plates containing coverslips. Cells were collected for lysate and coverslips were subjected to immunofluorescence protocol describe in Material and Methods. Coverslips were mounted and examined for localization of Cyc B1-GFP (green) and Flag-Tuberin or Flag- Tuberin_[1–677]_ (red) and Hoechst (blue) was used as a nucleic marker.
(TIF)

S4 Fig pH3 as a marker for mitotic index in cells transfected with Tuberin or Tuberin_[1–677]_ under normal or low nutrient conditions. HEK-293 cells were transiently transfected with Tuberin-WT or Tuberin_[1–677]_ expression vectors on plates containing coverslips. After 18–20 hrs the cells were subjected to 10% FBS (normal nutrients–panel A) or 0.5% FBS (low nutrients–panel B) conditions for 24 hrs. Cells were collected for lysate and coverslips were subjected to immunofluorescence protocol describe in Material and Methods. Coverslips were mounted and examined for pH3 staining (green) and Flag-Tuberin or Flag-Tuberin_[1–677]_ (red) and Hoechst (blue) was used as a nucleic marker.
(TIF)

S1 Raw images (PDF)
