# Supplementary figures and images for: Dissecting the roles of the Tuberin protein in the subcellular localization of the G2/M Cyclin, Cyclin B1
Source: PLoS One. 2022 Aug 10;17(8):e0272741. doi: 10.1371/journal.pone.0272741 (PMC9365131; doi:10.1371/journal.pone.0272741)

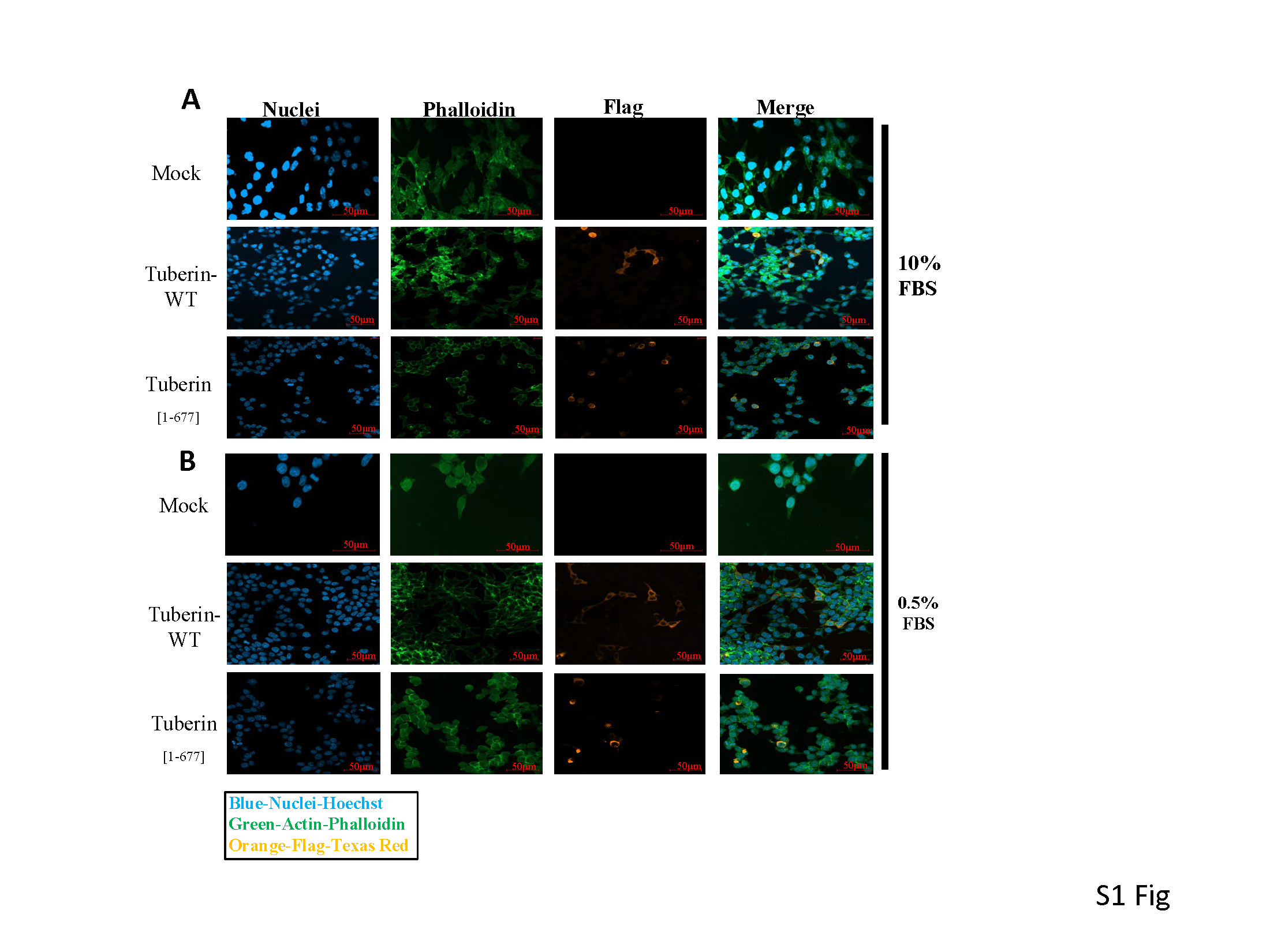

Supplement: S1 Fig — HEK-293 cells were transiently transfected with Tuberin WT or Tuberin[1–677] expression vectors on plates containing coverslips. After 18–20 hrs the cells were subjected to either 10% FBS (normal nutrient–panel A) or 0.5% FBS (low nutrient–panel B) conditions for 24 hrs. Cells were collected for lysate and coverslips were subjected to immunofluorescence protocol describe in Material and Methods. FLAG-Texas Red (red) labelling cells expressing Tuberin or Tuberin[1–677], Hoechst (blue) as a nuclei marker, and FITC-Phalloidin (green) to stain F-Actin. (TIF) [file pone.0272741.s001.tif]

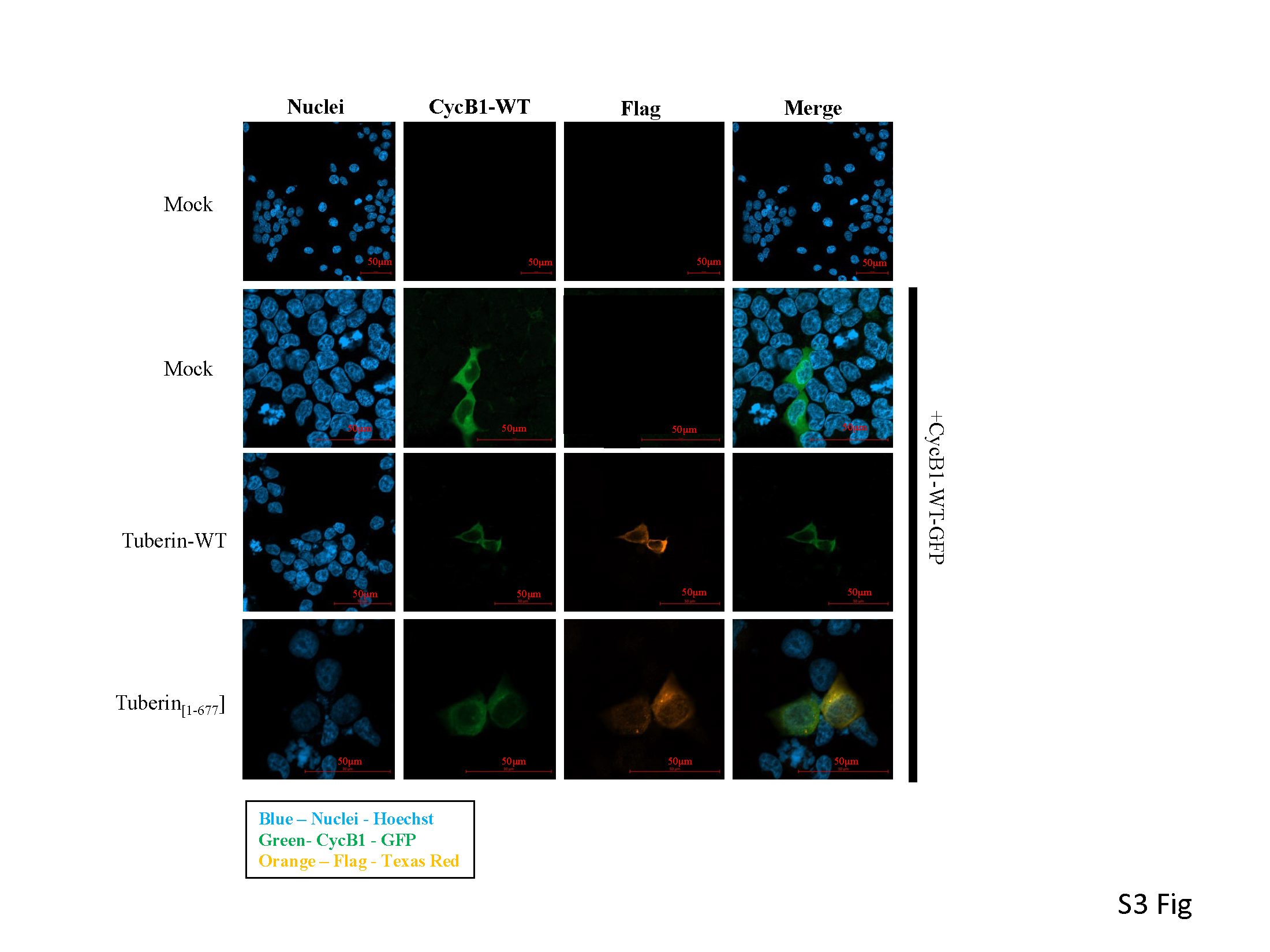

Supplement: S3 Fig — HEK-293 cells were transiently transfected with control (mock), Tuberin-WT or Tuberin[1–677] and CycB1 WT-GFP expression vectors on plates containing coverslips. Cells were collected for lysate and coverslips were subjected to immunofluorescence protocol describe in Material and Methods. Coverslips were mounted and examined for localization of Cyc B1-GFP (green) and Flag-Tuberin or Flag- Tuberin[1–677] (red) and Hoechst (blue) was used as a nucleic marker. (TIF) [file pone.0272741.s003.tif]

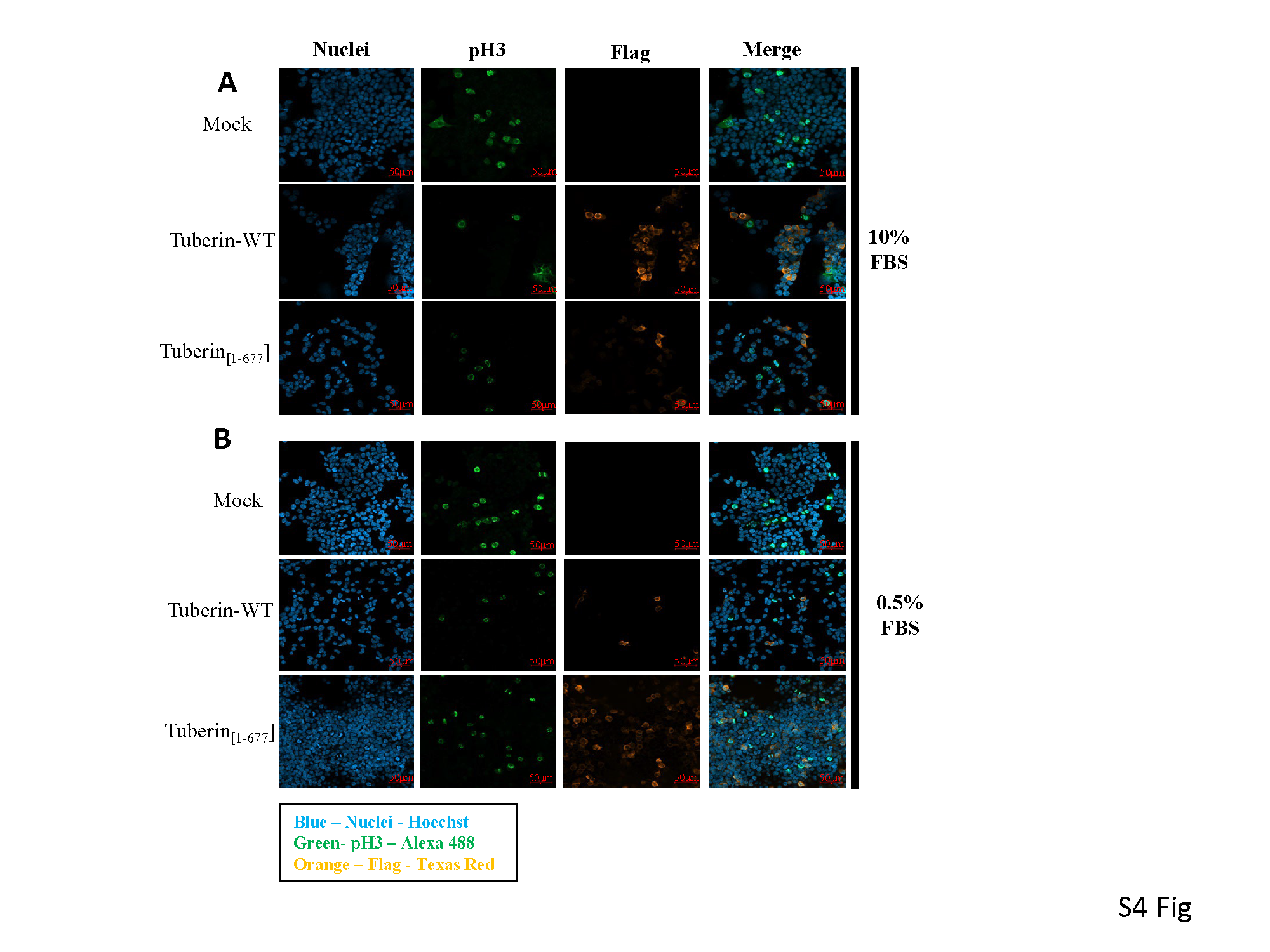

Supplement: S4 Fig — HEK-293 cells were transiently transfected with Tuberin-WT or Tuberin[1–677] expression vectors on plates containing coverslips. After 18–20 hrs the cells were subjected to 10% FBS (normal nutrients–panel A) or 0.5% FBS (low nutrients–panel B) conditions for 24 hrs. Cells were collected for lysate and coverslips were subjected to immunofluorescence protocol describe in Material and Methods. Coverslips were mounted and examined for pH3 staining (green) and Flag-Tuberin or Flag-Tuberin[1–677] (red) and Hoechst (blue) was used as a nucleic marker. (TIF) [file pone.0272741.s004.tif]
