# Supplementary material for: Dissecting the roles of the Tuberin protein in the subcellular localization of the G2/M Cyclin, Cyclin B1
Source: PLoS One. 2022 Aug 10;17(8):e0272741. doi: 10.1371/journal.pone.0272741 (PMC9365131; doi:10.1371/journal.pone.0272741)
Supplement: S1 Raw images — (PDF) [file pone.0272741.s005.pdf]

Fig 2B

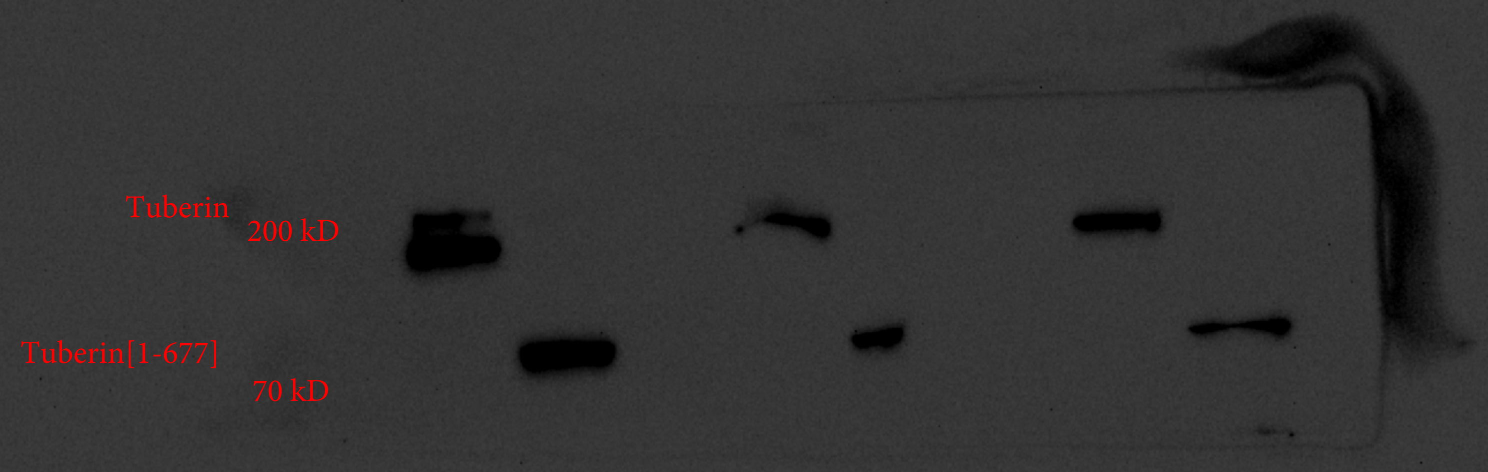

Fig 2B

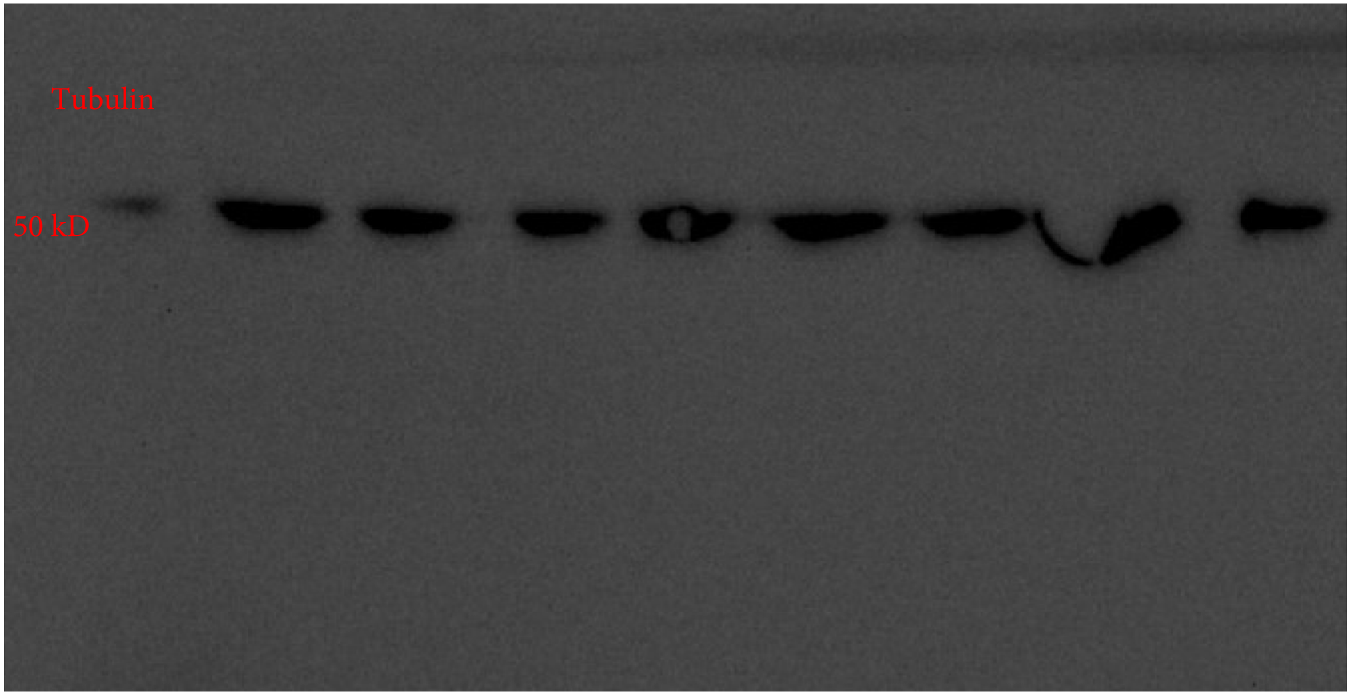

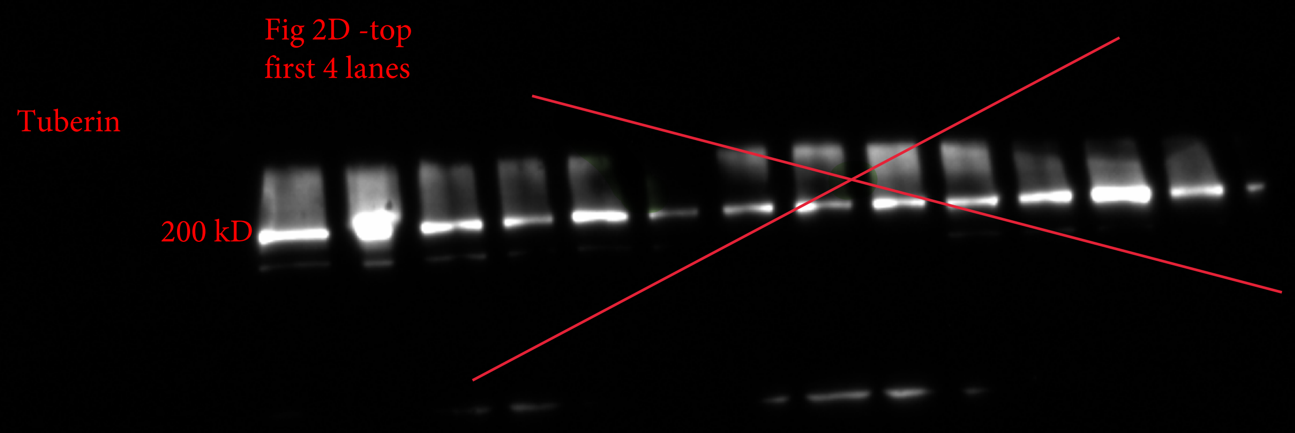
Fig 2D -top
first 4 lanes
Tuberin
200 kD

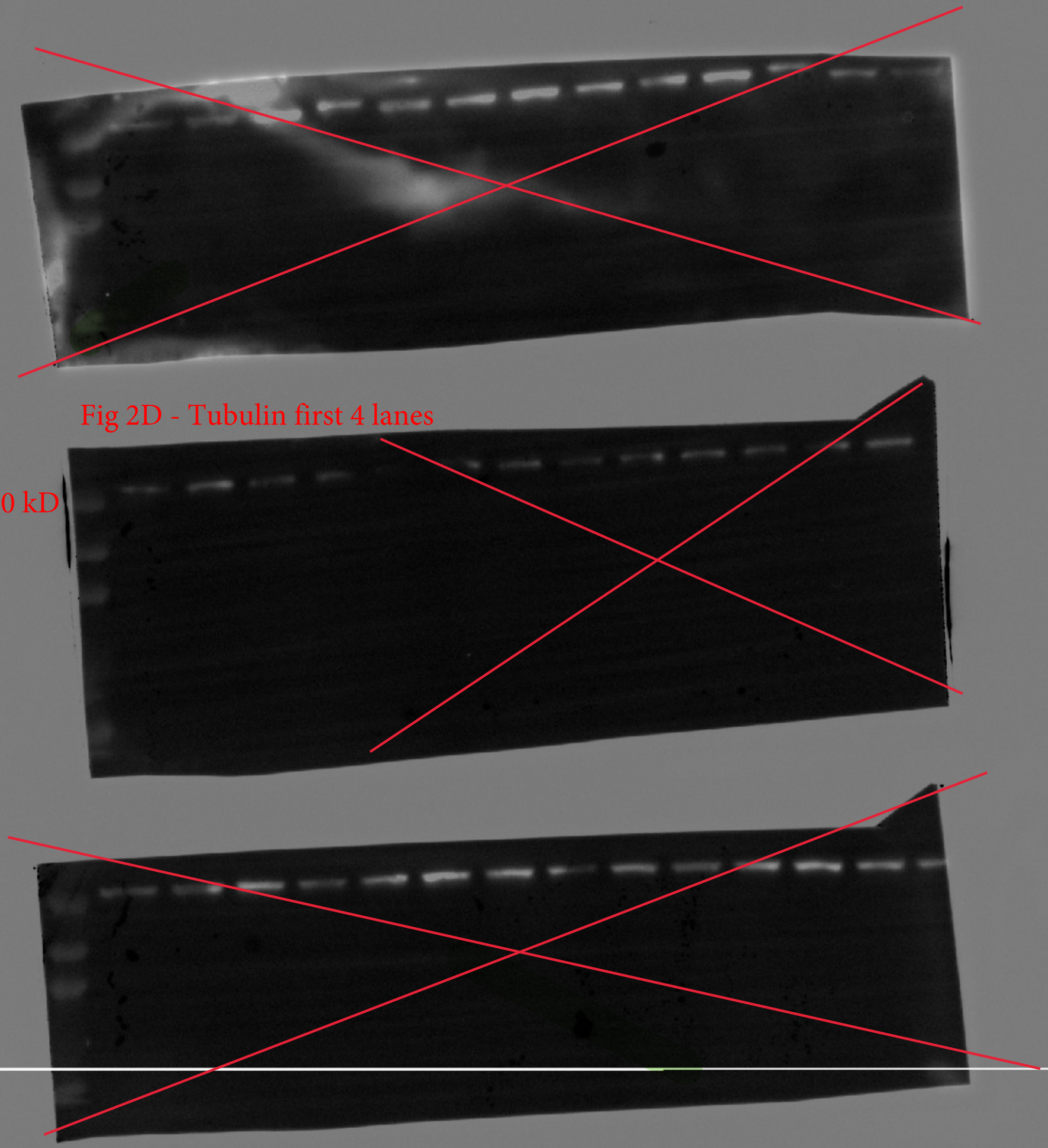

Tubulin for Tuberin lanes
Fig 2D - Tubulin first 4 lanes
Top
50 kD

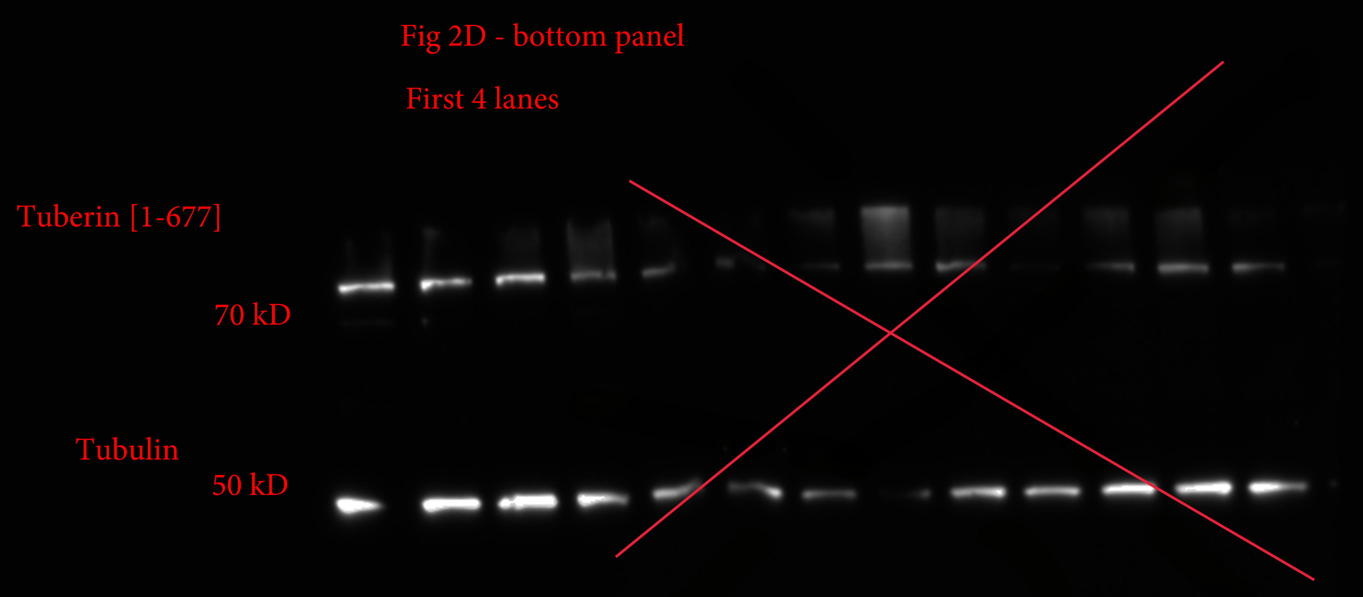
Fig 2D - bottom panel
First 4 lanes
Tuberin [1-677]
70 kD
Tubulin
50 kD
